# Association between obstructive sleep apnea and multiple adverse clinical outcomes: evidence from an umbrella review

**DOI:** 10.3389/fmed.2025.1497703

**Published:** 2025-03-17

**Authors:** Qian Wang, Hong Zeng, Jindong Dai, Ming Zhang, Pengfei Shen

**Affiliations:** Department of Urology, Institute of Urology, West China Hospital, Sichuan University, Chengdu, Sichuan, China

**Keywords:** obstructive sleep apnea, adverse clinical outcomes, umbrella review, meta-analysis, apnea-hypopnea index

## Abstract

**Background and objective:**

In recent years, there has been a notable rise in awareness regarding obstructive sleep apnea (OSA), and a significant number of potential OSA cases have been identified. Numerous studies have established associations between OSA and various adverse clinical outcomes. This umbrella review aims to summarize and evaluate the available evidence on the relationship between OSA and multiple adverse clinical outcomes.

**Methods:**

PubMed, Embase, and Web of Science databases were systematically searched from inception to September 2023. The AMSTAR and GRADE were used to evaluate the quality of meta-analysis literature and classify the quality of literature evidence. Furthermore, the size of the effect size of the association between OSA and adverse clinical outcomes were assessed by using either a random or fixed-effect model and 95% confidence interval (CI).

**Results:**

A total of 27 meta-analyses were enrolled with 43 adverse clinical outcomes. The umbrella review primarily reported the associations between sleep apnea syndrome and thyroid cancer (HR = 2.32,95%CI:1.35–3.98), kidney cancer (RR = 1.81, 95% CI: 1.20–2.74), liver cancer (RR = 1.19, 95% CI: 1.10–1.29), GERD (Gastroesophageal reflux disease)(OR = 1.53, 95% CI: 1.23–1.91), Atrial fibrillation (AF) (OR = 2.54, 95% CI: 2.20–2.92), osteoporosis (OR = 2.03, 95% CI: 1.26–3.27), and diabetes (OR = 1.40, 95% CI: 1.32–1.48). Overall, the AMSTAR rating scale and GRADE quality assessment included in the meta-analysis were generally low.

**Conclusion:**

Our study shows that OSA is significantly associated with a variety of adverse clinical outcomes, especially an increased risk of certain malignancies, and some adverse clinical outcomes are closely related to OSA severity.

## Introduction

Obstructive sleep apnea (OSA) is a very common sleep disorder characterized by intermittent narrowing of the upper respiratory tract (anatomic stenosis of the upper airway) (1). It usually presents with reduced airflow and loud snoring (2). Diagnosis of OSA relies on polysomnography monitoring, it is characterized by the cessation of inhaled airflow for a duration of 10 s or longer. Hypopnea, on the other hand, is defined as a reduction in inhaled airflow by at least 30% for a period of 10 s or more, accompanied by either a decrease in blood oxygen saturation or arousal from sleep. Severity can be assessed based on the apnea-hypopnea index (AHI: the number of apneas and hypopneas observed per hour) (3). In OSA patients, due to complete or partial obstruction of the upper respiratory tract, insufficient oxygen content in the body leads to hypoxemia, autonomic nerve fluctuation, and sleep disturbance, which causes various changes in the body and finally leads to the occurrence of adverse events (3, 4). In recent years, as awareness of OSA has grown, there has been a notable increase in the diagnosis of OSA. Current estimates indicate that approximately 34% of middle-aged men and 17% of middle-aged women are affected by this condition (5), and Up to 1 to 5% of otherwise healthy children are also affected by OSA (6, 7). Multiple studies have demonstrated the threat of OSA to human health.

A large number of systematic reviews and meta-analyses have been conducted summarizing the association of OSA with various adverse clinical outcomes and diseases. This evidence suggests an increased risk of cancer in the OSA group compared to the non-OSA group (8), including thyroid cancer (9), breast cancer (8), lung cancer (10), kidney cancer (8), liver cancer (8), and pancreatic cancer (8). We also found that the incidence of cancer was dose-dependent with the severity of OSA, the more severe OSA, the higher the incidence of cancer (11). At the same time, OSA also increased the incidence of metabolic syndrome (12), arterial hypertension (13), stroke (14), aortic dissection (15), eye disease (16, 17), periodontal disease (18), and other adverse events, and showed a dose relationship. While these meta-analyses have documented the associations between various systemic diseases and OSA, they primarily focus on the relationships between individual diseases and OSA.

Umbrella reviews can summarize the evidence for adverse events related to OSA, provide a more comprehensive assessment of quality and evidence credibility, and assess the potential bias in the relationship between exposure and outcomes, further improving the credibility of the evidence (19, 20). Therefore, we conducted this umbrella review to comprehensively summarize the results of the reported meta-analysis, to evaluate the association of OSA with various adverse events, and to provide guidance and advice for the prevention of OSA.

## Methods

### Umbrella review methods

We systematically retrieved, extracted, collated, and analyzed a large body of evidence on OSA and multiple adverse clinical outcomes from published meta-analyses and systematic reviews (21, 22). Systematic reviews with meta-analyses were included and those without meta-analyses were excluded. These outcomes are visually expressed in the form of forest maps. We have also registered this umbrella review on PROSPERO (CRD42023465621).

### Literature search

We have searched PubMed, Embase, and Web of Science database and systematically collected systematic reviews and meta-analyses of observational studies or randomized controlled trials from inception to September 2023. The strategy we used to retrieve systematic reviews and meta-analyses is as follows: (((((((((((((Apneas, Obstructive Sleep[Title/Abstract]) OR (Obstructive Sleep Apneas[Title/Abstract])) OR (Obstructive Sleep Apneas[Title/Abstract])) OR (Obstructive Sleep Apnea Syndrome[Title/Abstract])) OR (Obstructive Sleep Apnea[Title/Abstract])) OR (OSAHS[Title/Abstract])) OR (Syndrome, Sleep Apnea, Obstructive[Title/Abstract])) OR (Sleep Apnea Syndrome, Obstructive[Title/Abstract])) OR (Apnea, Obstructive Sleep[Title/Abstract])) OR (Sleep Apnea Hypopnea Syndrome[Title/Abstract])) OR (Syndrome, Obstructive Sleep Apnea[Title/Abstract])) OR (Upper Airway Resistance Sleep Apnea Syndrome[Title/Abstract])) OR (Syndrome, Upper Airway Resistance, Sleep Apnea[Title/Abstract])) AND ((((systematic review[Title/Abstract]) OR (meta-analysis[Title/Abstract])) OR (systematic overview[Title/Abstract]))). References of included studies were also searched manually. The article language is limited to English. Two authors (QW and MHW) independently conducted article searches and screened titles and abstracts already retrieved from the database and determined the final eligible meta-analysis by reading the full text. The disagreement about the literature search was eventually resolved by a third author (PFS).

### Eligibility criteria

This meta-analysis was considered adequate as long as articles reported associations between OSA and multiple adverse clinical outcomes. Whether it was a meta-analysis of observational studies (case–control studies, cohort studies, cross-sectional studies) or a meta-analysis of randomized controlled trials. We also did not limit the group, region, race, age, or gender in the article.

Articles should be excluded if they exist: (1) systematic reviews without meta-analysis; (2) research with incomplete data; (3) animal experiments; (4) writing in a language other than English. If there are multiple different adverse clinical outcomes in a meta-analysis article, the data for each health outcome is extracted separately and evaluated. If the meta-analysis article contains the same results, but the study type or population is different, each type of data is extracted and evaluated separately. If there are multiple articles reporting the same results, we will select the article with the largest sample size or the most recently published article, and exclude duplicate articles to avoid duplicate evaluation.

### Data extraction

The data in the eligible meta-analysis were extracted independently by two authors (QW and MHW) and evaluated for validation by two additional authors (HZ and JDD). From each meta-analysis, we collected: (1) name of the first author; (2) cancer or non-cancer outcomes; (3) exposure category [OSA/OSAS (obstructive sleep apnea syndrome)/OSASH (obstructive sleep apnea-hypopnea Syndrome)]; (4) number of articles included in the study; (5) type of study design (case–control/randomized control trial/cohort/cross-sectional study); (6) year of publication; (7) number of cases/total; (8) summary effect size (RR, relative risk; OR, odds ratio; HR, hazard risk with 95% CI); (9) type of effect model (fixed or random model); (10) heterogeneity (I^2^ statistic and Cochran’s Q test *p* value); (11) *p* value of Egger’s test or funnel plot. If there are analyses of special populations (pregnant women/post-operative patients), data will be extracted separately.

### Quality assessment of included studies and evidence

Two reviewers (QW and MHW) assessed the methodological quality of each included meta-analysis using AMSTAR (A Measurement Tool to Assess Systematic Reviews) (23, 24) (Supplementary Table 1B). For the assessment of the quality of evidence for each outcome measure, we used GRADE (Grading of Recommendations, Assessment, Development, and Evaluation) to make the assessment and graded it into “high,” “moderate,” “low,” or “very low” (25) (Supplementary Table 1A). In addition, we divided the outcome evidence into four categories according to the evidence classification standard: class I (convincing evidence), class II (highly suggestive evidence), class III (suggestive evidence), class IV (weak evidence), and NS (non-significant) (19, 26) (Table 1). Specific evaluation criteria are shown in Supplementary Table 1A.

**Table 1:** Evidence classification criteria.

Category of evidence	Detail standard
Convincing evidence (class I)	>1,000 cases; statistical significance at *p* < 0.000001 (random effects); no evidence of small study effects and excess significance bias; 95% prediction interval excluded null value; heterogeneity (I^2^ < 50%)
Highly suggestive evidence (class II)	>1,000 cases; statistical significance at *p* < 0.000001 (random effects)
Suggestive evidence (class III)	>1,000 cases and statistical significance at *p* < 0.001
Weak evidence (class IV)	Statistical significance at *p* < 0.05
Non-significant (NS)	Statistical significance at *p* > 0.05

### Data analysis

We reanalyzed the effect sizes (OR, RR, HR) and their 95% CIs in the included meta-analyses using either a random effect model or a fixed effect model. The analysis included cancer outcomes, non-cancer outcomes, specific populations, and the relationship between OSA severity and adverse clinical outcomes. According to AHI, OSA can be divided into the following types: mild OSA AHI 5–15/ h, moderate AHI 15–30/h, and severe OSA AHI > 30/h (27). The association with adverse clinical outcomes was further analyzed according to the severity of OSA. We also reanalyzed the I^2^ statistics, Cochran’s Q test and *p* value of Egger’s regression test of the included meta-analyses (28–30). If we cannot extract valid data from the meta-analysis for re-analysis, we extract the aggregated data as completely as possible and assess heterogeneity and publication bias. *p* value <0.10 was considered statistically significant in the heterogeneity test. For other applicable tests, *p* < 0.05 was considered statistically significant. If there are both cohort studies and case–control studies in the same article, we extract the data separately for reanalysis (31). If special populations were reported in the meta-analysis (pregnant women or post-operative patients), data were extracted separately for analysis and evaluation.

## Results

### Characteristics of the included meta-analysis

**Figure 1:**
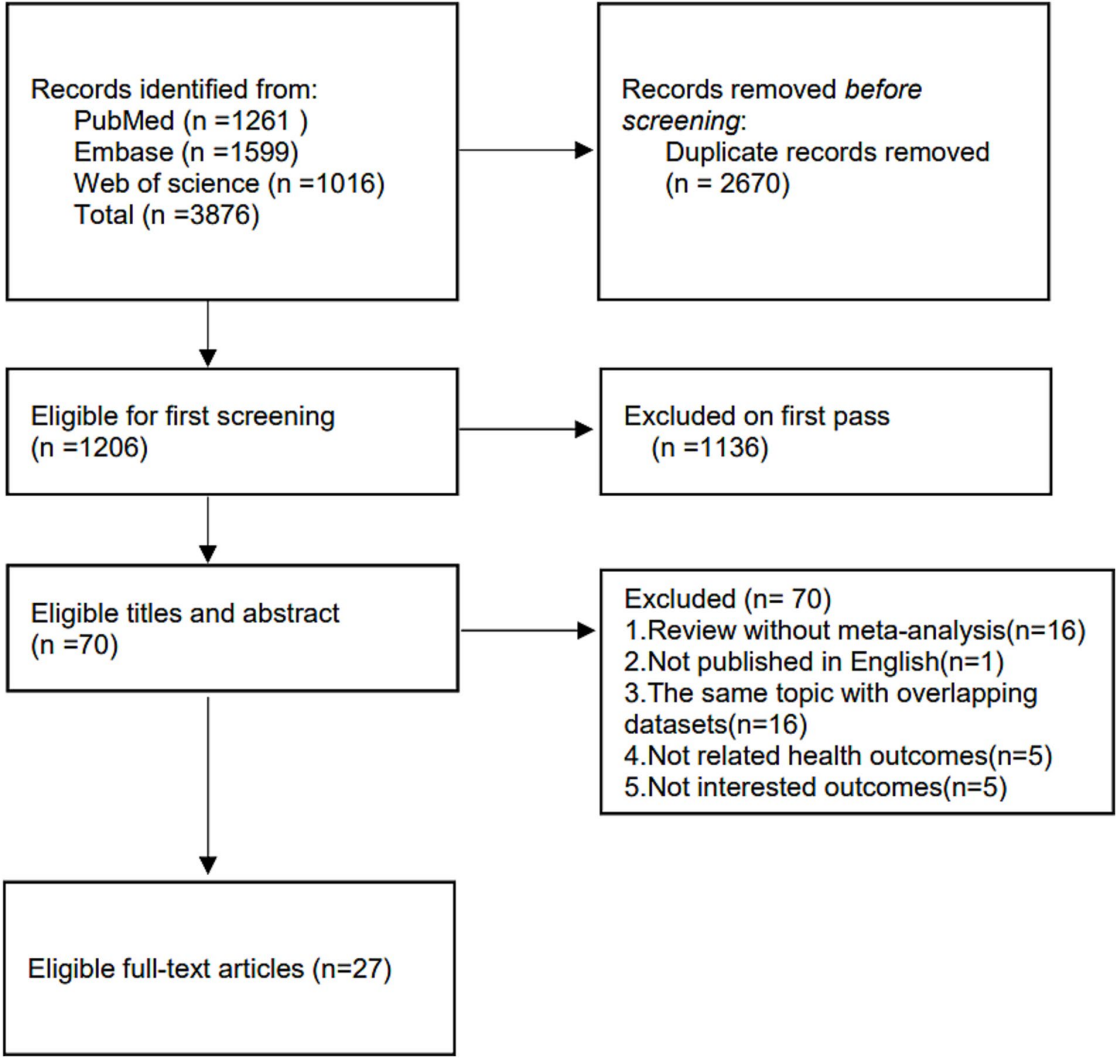
A detailed flowchart of literature selection.

The detailed flowchart of the literature selection is shown in Figure 1. Through systematic literature search, a total of 3,876 individual articles was identified and 27 meta-analyses were enrolled with 43 adverse clinical outcomes, including 11 cancer and 32 non-cancer outcomes (Tables 2, 3). Of these adverse clinical outcomes, nine are for special populations (pregnant women or post-operative patients), and eight are associated with the severity of OSA.

**Table 2:** Associations between OSA and non-cancer outcomes.

Outcome	Category	References	No. of cases/total	No. of studies	Cohort	Case- control	Cross- sectional	Meta metric	Effects model	Estimates	95%CI	I^2^;Q-test *p*-value	Egger’s test *p*-value
Significant associations													
Nocturia	OSA	Zhou et al. (15)	406/9,924	13	3	10	0	RR	Random	1.41	1.26–1.59	63.3%; 0.001	NA
Psoriasis	OSA	Ger et al. (39)	42,656/5,544,674	3	3	0	0	RR	Random	2.52	1.89–3.36	0.0%; 0.95	NA
Gastroesophageal refluxdisease (GERD)	OSA	Chehade et al. (32)	1,635/2,729	5	0	2	3	OR	Random	1.53	1.23–1.91	0.0%; 0.0001	NA
Periodontitis	OSA	Zhu et al. (18)	NA/8562	10	0	3	7	OR	Fixed	2.35	2.22–2.48	47.3%; 0.047	NA
Osteoporosis	OSAHS	Wang et al. (14)	5,701/112,304	3	3	0	0	OR	Random	2.03	1.26–3.27	57.0%; 0.10	NA
Type 2 diabetes mellitus (T2DM)	OSA	Qie et al. (36)	19,355/338912	16	16	0	0	OR	Fixed	1.40	1.32–1.48	40.8%; 0.045	NA
T2DM	Mild OSA	Yu et al. (45)	2,225/13,669	4	4	0	0	OR	Fixed	1.23	1.06–1.41	0.0%; 0.47	NA
T2DM	Moderate-to-severe OSA	Yu et al. (45)	923/3538	2	2	0	0	OR	Fixed	2.14	1.72–2.67	0.0%; 0.80	NA
Aortic dissection	Mild OSA	Zhou et al. (38)	16,019/55,911	3	1	2	0	OR	Fixed	1.60	1.01–2.53	0.0%; 0.44	0.575
Aortic dissection	Moderate-to-severe OSA	Zhou et al. (38)	103/257	2	0	2	0	OR	Fixed	4.43	2.59–7.59	0.0%; 0.62	NA
Metabolic syndrome	OSA	Xu et al. (12)	2,456/4,161	15	0	0	15	OR	Fixed	2.87	2.41–3.41	20.1%; 0.229	NA
Metabolic syndrome	OSA	Xu et al. (40)	1,156/1,560	5	0	5	0	OR	Fixed	2.56	1.98–3.31	35.6%; 0.184	NA
Metabolic syndrome	Mild OSA	Xu et al. (12)	NA	10	0	1	9	OR	Random	2.39	1.65–3.46	52.9%; 0.024	NA
Metabolic syndrome	Moderate-to-severeOSA	Xu et al. (40)	NA	10	0	4	6	OR	Random	3.42	2.28–5.13	64.2%; 0.003	NA
Atrial fibrillation	OSA	Zhang et al. (34)	187,928/528,300	12	12	0	0	OR	Random	2.54	2.20–2.92	68%; 0.0004	NA
Non-alcoholic fatty liver disease (NAFLD)	OSAS	Musso et al. (33)	NA	14	0	0	14	OR	Fixed	2.34	1.71–3.18	9.0%; 0.36	NA
Floppy eyelid syndrome (FES)	OSA	Aiello et al. (17)	153/708	7	7	0	0	OR	Fixed	3.41	2.03–5.73	0.0%; 0.52	NA
Lax eyelid condition (LEC)	OSA	Aiello et al (17)	431/1079	9	9	0	0	OR	Fixed	3.63	2.49–5.29	0.0%; 0.53	NA
Glaucoma	OSA	Bulloch et al. (16)	85,327/5,532,337	21	15	0	6	OR	Random	1.49	1.16–1.91	91%; 0.00001	NA
Keratoconus	OSA	Bulloch et al. (16)	1838/16,922	5	0	5	0	OR	Random	1.87	1.16–2.99	76%; 0.002	NA
Retinal vein occlusion (RVO)	OSA	Bulloch et al. (16)	215/35,898	4	0	4	0	OR	Fixed	2.71	1.83–4.00	19%; 0.30	NA
Essential hypertension	Mild OSA	Hou et al. (13)	NA/27414	11	3	0	8	OR	NA	1.18	1.09–1.27	NA; NA	0.380
Essential hypertension	Moderate OSA	Hou et al. (13)	NA/45113	17	4	0	13	OR	Fixed	1.32	1.20–1.43	3.2%; 0.416	0.001
Essential hypertension	Severe OSA	Hou et al. (13)	NA/25116	11	3	0	8	OR	Fixed	1.56	1.29–1.83	0.0%; 0.546	0.079
Resistant hypertension	OSA	Hou et al. (13)	NA/1465	6	0	6	0	OR	Fixed	2.84	1.70–3.98	0.0%; 0.816	0.078
Major adverse cardiac events (MACEs)	Moderate OSA	Xie et al. (44)	NA/14,542	6	6	0	0	RR	Fixed	1.16	1.01–1.33	0.0%; 0.597	0.052
Major adverse cardiac events (MACEs)	Severe OSA	Xie et al. (44)	NA/18,022	9	9	0	0	RR	Random	2.04	1.56–2.66	55.7%; 0.021	0.001
Stroke	Severe OSA	Wang et al. (35)	260/8,053	4	4	0	0	RR	Fixed	2.15	1.42–3.24	12.6%; 0.311	0.241
All-cause mortality	severe OSA	Wang et al. (51)	1404/12,091	6	6	0	0	RR	Fixed	1.92	1.38–2.69	37.5%; 0.156	0.044
Preterm birth	OSA	Xu et al. (12)	NA/722	3	3	0	0	RR	Fixed	1.90	1.24–2.91	48.2%; 0.145	NA
Cesarean delivery	OSA	Xu et al. (40)	NA/722	3	3	0	0	RR	Fixed	1.87	1.52–2.29	0%; 0.539	NA
NICU admission	OSA	Xu et al. (12)	NA/757	4	3	0	1	RR	Fixed	2.65	1.86–3.76	29.6%; 0.235	NA
Diabetic retinopathy	OSA	Zhu et al. (41)	NA/1,092	6	0	6	0	OR	Random	2.01	1.49–2.72	52.4%; 0.062	0.128
Postoperative respiratory complications.	OSA	Sun et al. (42)	16,719/55,637	19	19	0	0	OR	Random	1.91	1.54–2.36	51%; 0.006	0.5867
Postoperative cardiac complications.	OSA	Sun et al. (42)	1,238/27,120	18	0	0	0	OR	Random	1.74	1.25–2.42	67%; 0.0001	0.8803
Postoperative delirium	OSA	He et al. (43)	NA/1,218,393	16	15	1	0	OR	Random	3.73	2.34–5.96	60.7%; NA	NA
None-significant associations													
Gout	OSAS	Shi et al. (37)	3,814/154,455	3	3	0	0	HR	Random	1.25	0.91–1.70	91%; NA	0.876
Central serous chorioretinopathy (CSR)	OSA	Bulloch et al. (16)	453/33,3,709	2	0	2	0	OR	Random	2.28	0.65–7.97	72%; 0.06	NA
Idiopathic intracranial hypertension (IIH)	OSA	Bulloch et al. (16)	3050/2,490,100	4	1	3	0	OR	Random	1.29	0.33–5.01	91%; 0.00001	NA
Major adverse cardiac events (MACEs)	Mild OSA	Xie et al. (44)	NA/13,333	5	5	0	0	RR	Fixed	0.98	0.87–1.11	7.5%; 0.364	0.132
Coronary heart disease (CHD)	Severe OSA	Wang et al. (51)	967/6,266	3	3	0	0	RR	Random	1.21	0.75–1.96	52.6%; 0.097	NA
Glaucoma	OSA	Bullochet al (16).	26,545/3,278,272	12	12	0	0	OR	Random	1.50	0.93–2.45	95%; 0.00001	NA
Gestational diabetes	OSA	Xu et al. (40)	NA/692	4	3	0	0	RR	Random	1.40	0.62–3.19	56%; 0.078	NA

CI, confidence interval; RR, relative risk; OR, odds ratio; HR, hazard risk; NA, not available; OSA, obstructive sleep apnea; OSAHS, Obstructive Sleep Apnea-Hypopnea Syndrome; OSAS, obstructive sleep apnea syndrome; NICU, Neonatal intensive care unit.

**Table 3:** Associations between OSA and cancer outcomes.

Outcome	Category	References	No. of cases/total	No. of studies	Cohort	Case- control	Meta metric	Effects model	Estimates	95%CI	I^2^;Q-test *p*-value	Egger’s test *p*-value
Significant associations												
Cancer incidence	OSA	Wu et al. (8)	96,217/330611	11	10	1	RR	Fixed	1.42	1.29–1.55	32%; 0.15	0.27
Cancer incidence	mild OSA	Cheng et al. (11)	1728/30,973	5	5	0	RR	Fixed	1.14	1.04–1.25	0.0%; 0.95	0.113
Cancer incidence	moderate OSA	Cheng et al. (11)	1,654/27,519	5	5	0	RR	Fixed	1.36	1.24–1.50	11%; 0.35	0.189
Cancer incidence	severe OSA	Cheng et al. (11)	2,135/31,394	5	5	0	RR	Fixed	1.59	1.45–1.74	46%; 0.11	0.114
Thyroid cancer	OSA	Tan et al. (9)	NA/2,839,325	4	3	1	HR	Random	2.32	1.35–3.98	95%; 0.0001	NA
Kidney cancer	OSA	Wu et al. (8)	NA/939,116	3	2	1	RR	Random	1.81	1.20–2.74	90%; 0.01	NA
Breast cancer	OSA	Wu et al. (8)	NA/1,218,393	5	4	1	RR	Random	1.32	1.03–1.70	92%; 0.01	NA
Lung cancer	OSA	Cheong et al	22,855/4,885,518	4	4	0	HR	Random	1.25	1.02–1.53	97; 0.00001	NA
Liver cancer	OSA	Wu et al. (8)	NA/904,714	2	1	1	RR	Fixed	1.19	1.10–1.29	0%; 0.80	NA
Pancreatic cancer	OSA	Wu et al. (8)	NA/733,850	2	2	0	RR	Fixed	1.23	1.14–1.33	0%; 0.45	NA
Non-significant associations												
Prostate cancer	OSA	Wu et al. (8)	NA/939,116	3	2	1	RR	Random	1.09	0.83–1.44	90%; 0.01	NA
Bladder cancer	OSA	Wu et al. (8)	NA/904714	2	1	1	RR	Random	1.62	0.62–4.27	83%; 0.02	NA
Reproductive cancer	OSA	Wu et al. (8)	NA/733,850	2	2	0	RR	Random	1.80	0.77–4.23	98%; 0.01	NA
Colorectal cancer	OSA	Wu et al. (8)	NA/960,179	5	3	2	RR	Random	1.05	0.87–1.28	91%; 0.01	NA

CI, confidence interval; RR, relative risk; HR, hazard risk; NA, not available; OSA, obstructive sleep apnea.

**Figure 2:**
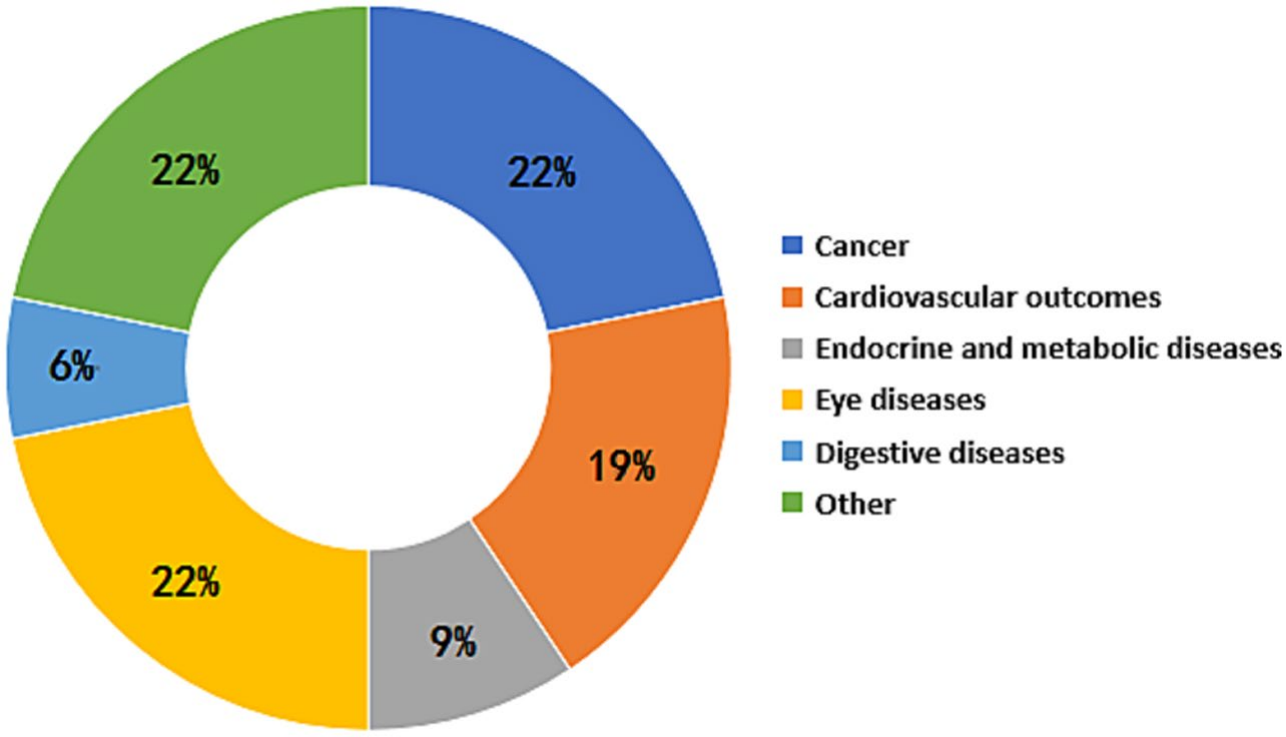
Map of adverse clinical outcomes associated with OSA.

The umbrella meta-analysis included primarily reported associations between sleep apnea syndrome and eye diseases (*n* = 7), endocrine and metabolic diseases (*n* = 5), cardiovascular diseases (*n* = 8), cancer outcomes (*n* = 11), digestive diseases (*n* = 2), adverse clinical outcomes for special populations (*n* = 8), adverse clinical outcomes associated with OSA severity (*n* = 16) (Figure 2). Among the 43 adverse clinical outcomes, 32 adverse clinical outcomes had significant relationships with OSA, 9 adverse clinical outcomes had no significant associations with OSA, and 2 adverse clinical outcomes had unclear relationships with OSA due to different study types.

### AMSTAR and GRADE evaluation of included meta-analyses

The quality of GRADE evidence for all adverse clinical outcomes was mostly low (26.3%) or very low (63.2%), and only 5 outcome measures, including psoriasis, Periodontitis, T2DM (Type 2 diabetes mellitus), metabolic syndrome and resistant arterial hypertension, had moderate quality evidence. In the AMSTAR score, all adverse clinical outcomes were scored between 7 and 10, with a median score of 8. Across all the adverse clinical outcomes we included, most of the evidence for adverse clinical outcomes fell into class III (suggestive evidence) (45.6%) or class IV (weak evidence) (35.1%) with 11 outcomes classified as NS (Non-significant) (19.3%) (Table 4). Of all the outcomes collected (including outcomes that differed by subgroup analysis and study type), we found 26 outcomes with Q test *p* < 0.10 and 3 outcomes with no Q test P reported. We also found that 30 outcomes reported low levels of heterogeneity (I^2^ < 50%).

**Table 4:** Assessments of AMSTAR scores and GRADE classification.

Outcome	Category	Author	Year	AMSTAR	GRADE	Category of evidence
Cancer outcomes						
Cancer incidence	Mild OSA	Cheng	2021	8	Very low	IV
Cancer incidence	Moderate OSA	Cheng	2021	8	Very low	III
Cancer incidence	Severe OSA	Cheng	2021	8	Very low	III
Cancer incidence	OSA	Wu	2023	9	Low	IV
Kidney cancer	OSA	Wu	2023	8	Very low	IV
Breast cancer	OSA	Wu	2023	8	Very low	IV
Liver cancer	OSA	Wu	2023	8	Low	IV
Pancreatic cancer	OSA	Wu	2023	8	Low	IV
Prostate cancer	OSA	Wu	2023	8	Very low	NS
Bladder cancer	OSA	Wu	2023	8	Very low	NS
Reproductive cancer	OSA	Wu	2023	8	Very low	NS
Colorectal cancer	OSA	Wu	2023	8	Very low	NS
Thyroid cancer	OSA	Cheong	2022	9	Very low	III
Lung cancer	OSA	Tan	2022	9	Very low	IV
Non-cancer outcomes						
Nocturia	OSA	Zhou	2020	7	Very low	III
Psoriasis	OSA	Ger	2020	7	Moderate	III
GERD	OSA	Chehade	2023	10	Very low	III
Periodontitis	OSA	Zhu	2023	7	Moderate	IV
Osteoporosis	OSAH	Wang	2022	9	Low	IV
T2DM	OSA	Qie	2020	9	Moderate	IV
T2DM	Mild OSA	Yu	2021	9	Very low	III
T2DM	Moderate-to-severe OSA	Yu	2021	9	Very low	III
Aortic dissection	Mild OSA	Zhou	2018	8	Very low	IV
Aortic dissection	Moderate-to-severe OSA	Zhou	2018	8	Very low	III
Metabolic syndrome	OSA	Xu	2015	9	Moderate	IV
Metabolic syndrome	OSA	Xu	2015	9	Moderate	IV
Metabolic syndrome	Mild OSA	Xu	2015	9	Low	IV
Metabolic syndrome	Moderate-to-severe OSA	Xu	2015	9	Very low	IV
Atrial fibrillation	OSA	Zhang	2022	8	Low	IV
Non-alcoholic fatty liver disease	OSA	Musso	2013	10	Low	IV
Floppy eyelid syndrome	OSA	Aiello	2023	9	Very low	IV
Lax eyelid condition	OSA	Aiello	2023	9	Very low	IV
Glaucoma	OSA	Bulloch	2023	8	Very low	IV
Glaucoma	OSA	Bulloch	2023	8	Very low	NS
Keratoconus	OSA	Bulloch	2023	8	Very low	IV
Retinal vein occlusion	OSA	Bulloch	2023	8	Very low	III
Central serous chorioretinopathy	OSA	Bulloch	2023	8	Very low	NS
Idiopathic intracranial hypertension	OSA	Bulloch	2023	8	Very low	NS
Essential hypertension	Mild OSA	Hou	2018	9	Low	IV
Essential hypertension	Moderate OSA	Hou	2018	9	Very low	IV
Essential hypertension	Severe OSA	Hou	2018	9	Low	IV
Resistant hypertension	OSA	Hou	2018	9	Moderate	IV
Major adverse cardiac events	Mild OSA	Xie	2017	9	Low	NS
Major adverse cardiac events	Moderate OSA	Xie	2017	9	Low	IV
Major adverse cardiac events	Severe OSA	Xie	2017	9	Low	III
Stroke	Severe OSA	Wang	2013	9	Very low	III
All-cause mortality	Severe OSA	Wang	2013	9	Very low	III
Coronary heart disease	Severe OSA	Wang	2013	8	Very low	NS
Preterm birth	OSA	Xu	2014	8	Low	IV
Cesarean delivery	OSA	Xu	2014	8	Low	III
NICU admission	OSA	Xu	2014	8	Low	III
Gestational diabetes	OSA	Xu	2014	8	Very low	NS
Diabetic Retinopathy	OSA	Zhu	2017	9	Very low	IV
Postoperative respiratory complications	OSA	Sun	2022	9	Very low	III
Postoperative cardiac complications.	OSA	Sun	2022	9	Very low	III
Postoperative delirium	OSA	He	2022	8	Very low	III
Gout	OSAS	Shi	2019	8	Very low	NS

OSA, obstructive sleep apnea; OSAS, obstructive sleep apnea syndrome; AMSTAR, a measurement tool to assess systematic reviews; GRADE, Grading of Recommendations Assessment, Development, and Evaluation; T2DM,Type 2 diabetes mellitus; GERD, Gastroesophageal reflux disease; NS, Non-significant.

### Cancer outcomes associated with OSA

**Figure 3:**
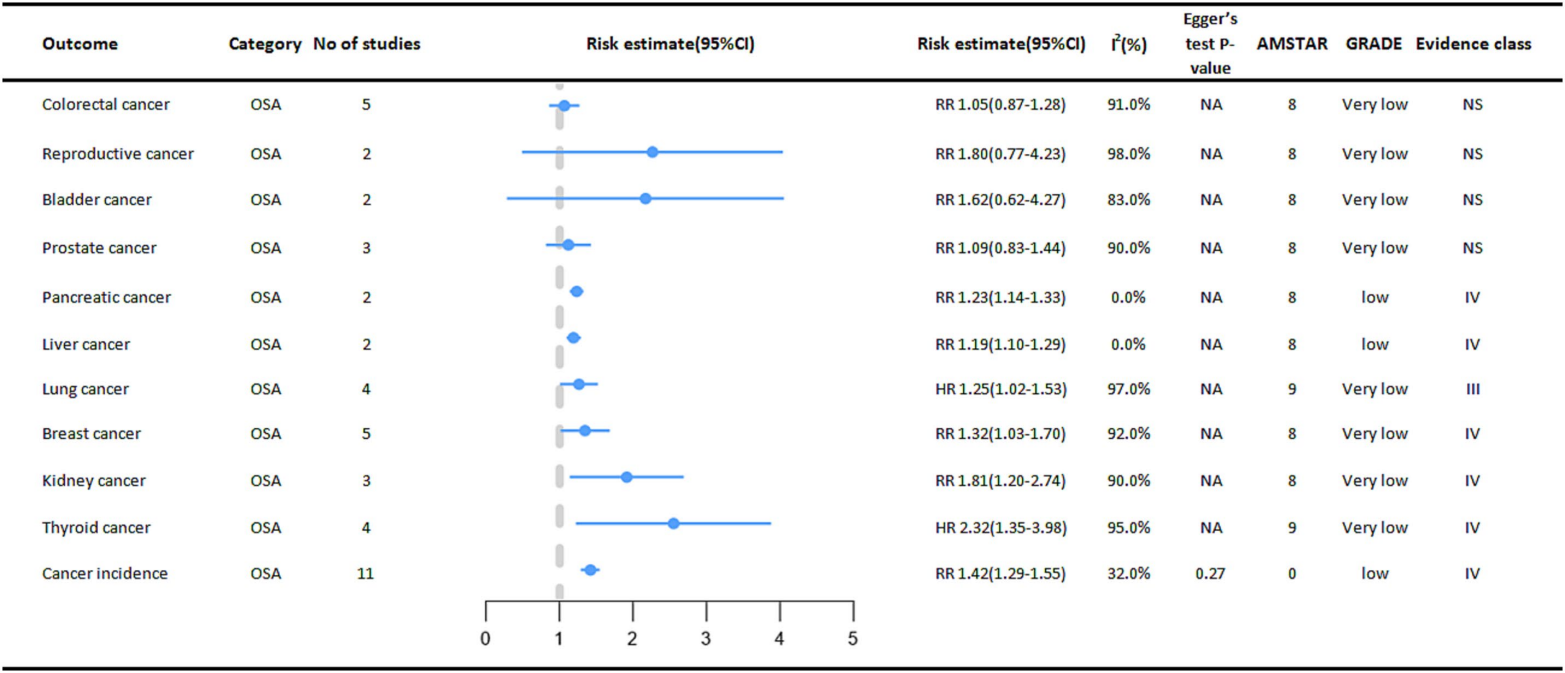
Pooled estimates of meta-analyses between OSA and cancer outcomes. The solid line represents the effect size and 95% confidence interval for each meta-analysis. CI, confidence interval; RR, relative risk; HR, hazard ratio; AMSTAR, a measurement tool to assess systematic reviews; GRADE, grading of recommendations assessment, Development and evaluation; NA, not available.

In the included meta-analysis, we collected 11 cancer outcomes associated with OSA (Figure 3). Overall, OSA is associated with an increased risk of pancreatic cancer, liver cancer, lung cancer, breast cancer, kidney cancer, and thyroid cancer. The OSA group exhibited a 42% higher overall cancer incidence (RR = 1.42, 95% CI: 1.29–1.55) compared to the control group (non-OSA). In subgroup analyses stratified by sex, a statistically significant difference in cancer incidence was observed among females (RR = 1.27, 95% CI: 1.06–1.51) (8). A meta-analysis also suggested that OSA significantly increases the risk of thyroid cancer (HR = 2.32, 95%CI:1.35–3.98), and subgroup analysis of studies followed for at least 5 years showed a more significant association between OSA and thyroid cancer incidence (HR = 3.27, 95% CI:2.80–3.82) (9).

We also found that OSA was significantly associated with a higher risk of kidney cancer (RR = 1.81, 95% CI: 1.20–2.74), higher risk of breast cancer (RR = 1.32, 95% CI: 1.03–1.70), higher risk of pancreatic cancer in cohort studies (RR = 1.23, 95% CI: 1.14–1.33), and higher risk of liver cancer (RR = 1.19, 95% CI: 1.10–1.29) (8).

Another study also found that OSA also increases the risk of lung cancer (HR = 1.25, 95% CI: 1.02–1.53), and subgroup analyses of studies followed for at least 5 years showed a more significant association between OSA and the risk of developing lung cancer (HR = 1.32, 95% CI: 1.27–1.37) (10).

### Non-cancer outcomes associated with OSA

Thirty-two non-cancer outcomes were also observed to be associated with OSA, of which 27 outcomes were significantly associated with OSA.

In digestive diseases, a meta-analysis of five studies showed that OSA increased the incidence of Gastroesophageal reflux disease (GERD) by 53% (OR = 1.53, 95% CI: 1.23–1.91) (32). OSA was significantly associated with an increased incidence of Non-alcoholic fatty liver disease (NAFLD), and subgroup analyses showed that OSA was associated with an increased risk of advanced (stage F3-4) fibrosis and non-alcoholic steatohepatitis (33).

**Figure 4:**
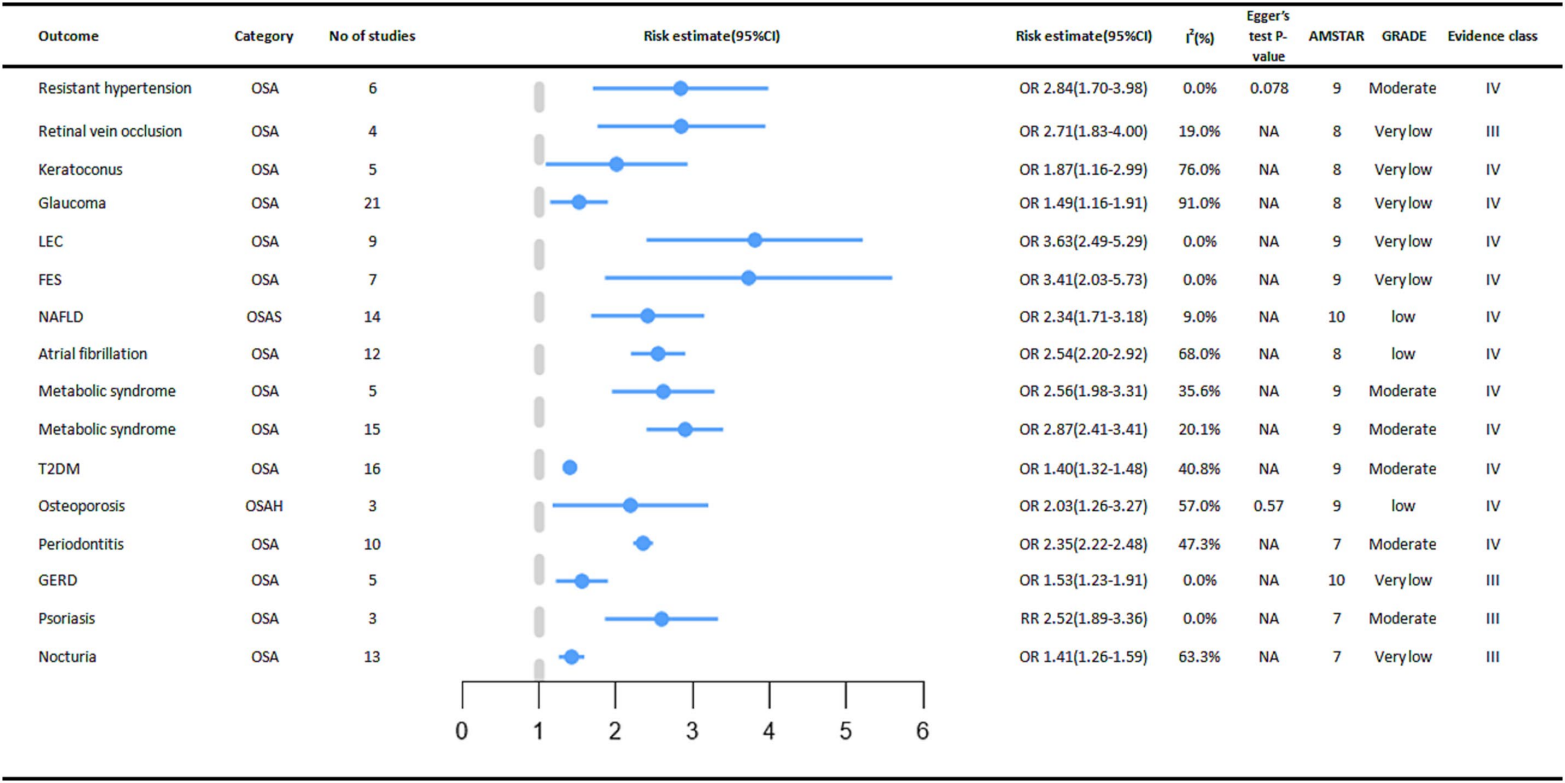
Significant pooled estimates of meta-analyses between OSA and Non-cancer outcomes. The solid line represents the effect size and 95% confidence interval for each meta-analysis. CI, confidence interval; RR, relative risk; OR, odds ratio; AMSTAR, a measurement tool to assess systematic reviews; GRADE, grading of recommendations assessment, Development and evaluation; NA, not available; LEC, Lax eyelid condition; FES, Floppy eyelid syndrome; NAFLD, Non-alcoholic fatty liver disease; T2DM, diabetes mellitus type 2; GERD, Gastro Esophageal Reflux Disease.

A positive association between OSA and cardiovascular outcomes was reported in multiple meta-analyses. A meta-analysis showed that people in the OSA group had a higher incidence of Atrial fibrillation (AF) (OR = 2.54, 95% CI: 2.20–2.92) (34). Subgroup analysis also showed an increased incidence in the OSA group including lone AF (OR = 1.71, 95% CI: 1.37–2.13), coronary artery bypass grafting (OR = 2.65, 95%CI: 2.32–3.01) and AF after ablation treatment (OR = 2.93, 95% CI: 2.47–3.49). Linear analysis showed a 1.26% increased risk of AF occurrence (95% CI 0.86–1.67%, *p* < 0.05) when AHI increased by 1 event per hour (34). There was a 1.84-fold increased risk of treatment-resistant arterial hypertension in the OSA population compared to the Non-OSA population (OR = 2.84, 95% CI: 1.70–3.98), and the increased risk was more prominent in the Caucasian population in the subgroup analysis (OR = 4.41, 95% CI: 1.84–6.98) (13) (Figure 4).

In terms of endocrine and metabolic diseases, we found that OSA also has a certain relationship with their occurrence. A meta-analysis of three cohort studies showed a significant association between OSA and an increased incidence of osteoporosis (OR = 2.03, 95% CI: 1.26–3.27) (35). Subgroup analysis showed a 90 percent increased risk of bone thinning in men with OSA (OR = 1.90, 95% CI: 1.33–2.72) and an increased risk of osteoporosis in women with OSA (OR = 2.56, 95% CI: 1.96–3.34). By age stratification, both the OSA group in older (> 65 years old) adults (OR = 2.62, 95% CI: 1.86–3.71) and the OSA group in middle-aged (40–65 years old) adults (OR = 1.73, 95% CI: 1.31–2.28) had an increased risk of osteoporosis (35) In a meta-analysis that included 16 cohort studies, the association between OSA and type 2 diabetes mellitus was reported, with a higher prevalence of type 2 diabetes mellitus in the OSA group compared to the control group (OR = 1.40, 95% CI: 1.32–1.48) (36). In addition, in a meta-analysis that included 15 cross-sectional or case–control studies, the presence of OSA increased the incidence of metabolic syndrome, respectively (OR = 2.87, 95% CI: 2.41–3.41) and (OR = 2.56, 95% CI: 1.98–3.31) (12). The relationship between the occurrence of gout and OSA was not significant and there was no statistical significance (37).

In the meta-analysis we collected, seven eye diseases were associated with OSA. Compared to people without OSA, OSA increases the incidence of these eye diseases for example FES (Floppy eyelid syndrome) (OR = 3.41, 95% CI: 2.03–5.73) (17), LEC (Lax eyelid condition) (OR = 3.63, 95% CI: 2.49–5.29) (17), Glaucoma (OR = 1.49, 95% CI: 1.16–1.91) (16), Keratoconus (OR = 1.87, 95% CI: 1.16–2.99) (16), RVO (Retinal vein occlusion) (OR = 2.71, 95% CI: 1.83–4.00) (16). However, the relationships between OSA and CSR (Central serous chorioretinopathy), IIH (Idiopathic intracranial hypertension), and Glaucoma (only one case–control study was included) were not significant (16).

The presence of OSA increased the risk of nocturia by 41% (RR = 1.41, 95% CI: 1.26–1.59). Subgroup analysis stratified by sex showed a statistically significant association between OSA and the risk of nocturia in men (RR = 1.49, 95% CI: 1.09–2.03) (38). A meta-analysis of 3 cohort studies found that OSA was associated with an increased incidence of psoriasis (RR = 2.52, 95% CI: 1.89–3.36). We also found a significantly increased risk of OSA in psoriasis patients (OR = 2.60, 95% CI: 1.07–6.32), with a bidirectional association (39). In a meta-analysis of 10 studies, we found an increased prevalence (OR = 2.35, 95% CI: 2.22–2.48) of periodontitis in the OSA population compared to the control group (18).

Relationship between OSA and multiple adverse clinical outcomes in specific populations.

**Figure 5:**
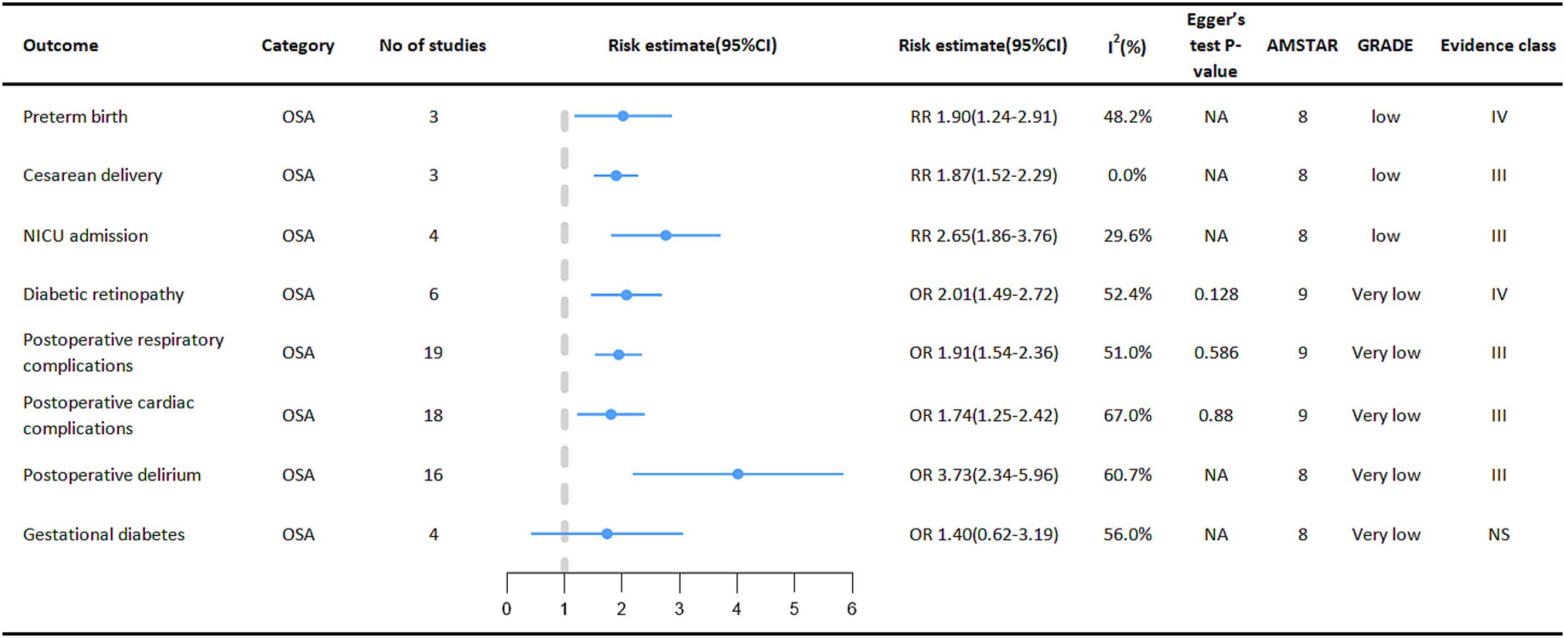
Pooled estimates of meta-analyses between OSA and multiple adverse clinical outcomes in a specific population. The solid line represents the effect size and 95% confidence interval for each meta-analysis. CI, confidence interval; RR, relative risk; OR, odds ratio; AMSTAR, a measurement tool to assess systematic reviews; GRADE, grading of recommendations assessment, Development and evaluation; NA, not available; NICU, neonatal intensive care unit.

Special groups of people with OSA deserve our attention (Figure 5). One meta-analysis showed that OSA in pregnant women increased the risk of perinatal adverse maternal and infant outcomes. Outcomes showed that pregnant women with OSA were associated with an increased probability of neonatal preterm birth (RR = 1.90, 95% CI: 1.24–2.91), cesarean section delivery (RR = 1.87, 95% CI: 1.52–2.29), and admission to the NICU (Neonatal intensive care unit) (RR = 2.65, 95% CI: 1.86–3.76). However, the relationship between OSA and gestational diabetes mellitus in pregnant women was not significant (40). At the same time, among diabetic patients, the incidence of diabetic retinopathy is higher in the OSA group (OR = 2.01, 95% CI: 1.49–2.72) (41).

Surgical patients are also a focus of concern, with patients with OSA having a higher incidence of various adverse events during the perioperative period. Outcome measures showed a higher risk of postoperative cardiac disease complications (OR = 1.74, 95% CI: 1.25–2.42) (42), respiratory complications (OR = 1.92, 95% CI: 1.54–2.36) (42) and postoperative delirium (OR = 3.73, 95% CI: 2.34–5.96) (43) in the OSA population than in the Non-OSA population.

### Adverse clinical outcomes associated with OSA severity

**Figure 6:**
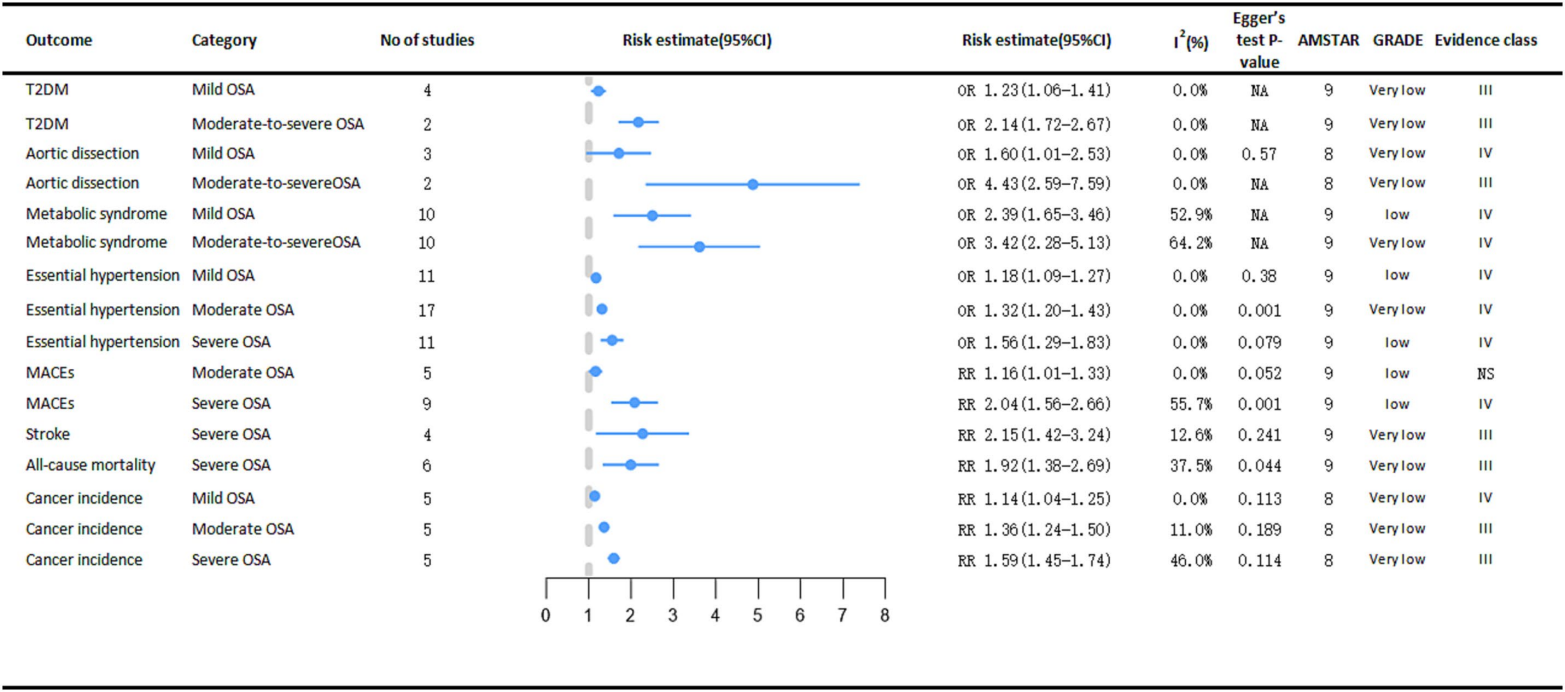
Significant pooled estimates of meta-analyses between OSA and adverse clinical outcomes associated with OSA severity. The solid line represents the effect size and 95% confidence interval for each meta-analysis. CI, confidence interval; RR, relative risk; OR, odds ratio; AMSTAR, a measurement tool to assess systematic reviews; GRADE, grading of recommendations assessment, Development and evaluation; NA, not available; T2DM, diabetes mellitus type 2; MACEs, major adverse cardiac events.

Depending on AHI, OSA can be classified as mild, moderate, or severe (27). The risk of developing cancer varies according to the severity of Obstructive Sleep Apnea (OSA) (Figure 6). The meta-analysis results demonstrated a significant association between OSA severity, as stratified by the Apnea-Hypopnea Index (AHI), and an elevated risk of various types of cancer. Specifically, compared with non-OSA patients, those with mild OSA (RR = 1.14, 95% CI: 1.04–1.25), moderate OSA (RR = 1.36, 95% CI: 1.24–1.50), and severe OSA (RR = 1.59, 95% CI: 1.45–1.74) exhibited progressively higher rates of cancer incidence, which increased in tandem with the severity of OSA (11).

Severe OSA is significantly associated with cardiogenic shock (RR = 2.15, 95% CI: 1.42–3.24) and cardiac all-cause mortality (RR = 1.92, 95% CI: 1.38–2.69) (14). As OSA became more severe, the risk of essential arterial hypertension also increased, from mild to moderate to severe, the risk of essential arterial hypertension increased by 18% (OR = 1.18, 95% CI: 1.09–1.27), 32% (OR = 1.32, 95% CI: 1.20–1.43), and 56% (OR = 1.56, 95% CI: 1.29–1.83), respectively (13). Analyses revealed a significant positive correlation between moderate OSA (OR = 1.16, 95% CI: 1.01–1.33) and severe OSA (OR = 2.04, 95% CI: 1.56–2.66) and the risk of major adverse cardiac events (MACEs). In contrast, mild OSA did not demonstrate a statistically significant impact on the risk of MACEs (44).

A meta-analysis encompassing two case–control studies demonstrated a significantly stronger association between moderate-to-severe obstructive sleep apnea (OSA) and aortic dissection (OR = 4.43, 95% CI: 2.59–7.59) compared to mild OSA (OR = 1.60, 95% CI: 1.01–2.53) (15). In a meta-analysis of metabolic diseases, people with moderate to severe OSA had a higher risk of both T2DM (moderate to severe OSA OR = 2.14, 95% CI: 1.72–2.67; mild OSA OR = 1.23, 95% CI: 1.06–1.41) (45) and metabolic syndrome (moderate to severe OSA OR = 3.42, 95% CI: 2.28–5.13; mild OSA OR = 2.39, 95% CI: 1.65–3.46) (12) than people with mild OSA. However, the increased risk of coronary artery disease (CAD) was not statistically significant in people with severe OSA (14).

## Discussion

OSA has been linked to multiple adverse clinical outcomes in multiple meta-analyses. The results showed that OSA was positively associated with the risk of cardiovascular disease and eye diseases. In addition, a significant harmful relationship between OSA and diseases of the endocrine and metabolic systems including osteoporosis (35), T2DM (36), and metabolic syndrome (12) were observed. At the same time, there were significant harmful correlations between OSA and nocturia (38), psoriasis (39), GERD (32), periodontitis (18), and NAFLD (33). We observed that OSA was able to significantly increase their risk of cancer incidence (8), thyroid cancer (9), kidney cancer (8), breast cancer (8), lung cancer (10), liver cancer (8), and pancreatic cancer (8). Compared with non-OSA patients, the incidence of cancer in patients with mild, moderate, and severe OSA increased with the severity of OSA (11).

In the perinatal population of pregnant women, we observed that pregnant women in the OSA group had a higher risk of multiple adverse clinical outcomes. OSA will affect the changes in the pregnant woman’s body and thus have a great impact on the pregnant woman herself and the fetus, mainly because the developing uterus pushes the abdominal contents upward, thus affecting the respiratory system (46). This is especially true in late and obese pregnancy (46, 47). The rate of cesarean section and intensive care unit occupancy in newborns increased due to OSA, induced hypoxemia, hypercapnia, systemic inflammatory response, and endothelial dysfunction in pregnant women. Most of the reasons for staying in the NICU were secondary respiratory illness, which was particularly evident in obese pregnant women (40, 48). Patients with OSA had an approximately 2-fold increased risk of postoperative respiratory complications, including respiratory failure, pneumonia, and tracheal intubation, and cardiovascular complications, including heart failure, atrial fibrillation, and myocardial infarction (42). The possible mechanism is that OSA is associated with postoperative hemodynamic instability. Subgroup analysis showed that OSA patients who underwent cardiac surgery and non-cardiac surgery had an increased risk of postoperative cardiac complications (49, 50). Due to the hypoxic state of OSA, activated oxidative stress, impaired blood–brain barrier, and reduced expression of brain-derived neurotrophic factor, adult neurogenesis, and sirtuin1 in the hippocampus may be associated with postoperative delirium in perioperative OSA patients (43, 51). There was a higher incidence of OSA in diabetic patients, and OSA increases the risk of retinopathy in diabetic patients, both of which have been proven to have a bidirectional relationship (52). The possible mechanism is that OSA aggravates autonomic dysfunction and microangiopathy in diabetic patients (53).

Cancer is a disease with a very complex etiology, and the mechanism by which OSA affects the increased risk of cancer is not fully understood. Most *in vitro* experiments have shown that OSA causes intermittent hypoxia in the body, leading to tumor growth, angiogenesis, and tumor metastasis (54–56). OSA increased the risk of liver cancer by 19%, and the mechanism may be that intermittent hypoxia increased the expression of vascular endothelial growth factor (VEGF) in liver cancer cells and the potential to promote angiogenesis. *In vitro* and *in vivo* model studies showed that although intermittent limping did not promote kidney tumor growth, IH significantly increased endothelial cells and circulating VEGF in vivo models (*p* < 0.001). In thyroid cancer, chronic hypoxia within the tumor stimulates the production of hypoxia-inducing factors, thereby regulating key genes such as vascular endothelial growth factor, GLUT-1 (glucose transporter 1), P21, and carbonic anhydrase 9 to jointly promote tumor growth, invasion, and metastasis (57, 58). There are several different theories about the mechanism of OSA and lung cancer. One hypothesis is that OSA leads to sleep fragmentation, increased sympathetic activation, and systemic inflammatory states (such as elevated C-reactive protein) (59–62). Another hypothesis is that intermittent hypoxia induces upregulation of hypoxia-inducing factors, which promotes the occurrence and development of tumors.

Pathologic nocturia is often associated with prostate disease and bladder overactivity, OSA is also currently considered to be one of the risk factors for nocturia, and it is proposed that nocturia is an independent predictor for the diagnosis of sleep disorders in men (63). The bidirectional association between OSA and psoriasis suggests a common systemic inflammatory pathway (64) The activity of IL-7, TNF, IL-6, and C-reactive protein was significantly increased in OSA patients, and the increase of this activity was also significantly correlated with psoriasis (65, 66). During apnea episodes at night, gastroesophageal gradient pressure increases due to increased negative pressure in the chest cavity, causing acid reflux. At the same time, due to the exposure of stomach acid, directly or indirectly through the vaso-vagal reflex, the upper respiratory tract sensitivities resulted in obstructive symptoms (67, 68). In cell and animal models, intermittent hypoxia promotes hepatic triglyceride accumulation, necrotic inflammation, and fibrosis by activating responses including nuclear factor-KB, hypoxia-inducible factors, and unfolded proteins, which are possible mechanisms of OSA and NAFLD (69). It has been reported that oral respiration in OSA patients reduced oral moisture, thereby reducing the self-cleaning ability of saliva, leading to bacterial colonization of the periodontal (70). Endocrine and metabolic problems are often associated with OSA in both directions. OSA may increase the risk of osteoporosis, the possible mechanisms are that OSA leads to vitamin D deficiency and secondary to hyperparathyroidism leading to osteoporosis. In addition, OSA patients had a 40% increased risk of T2DM, and there was also a dose–response relationship, with an 8% increased risk of T2DM for every 5 times /h increase in AHI value (36). The relationship between OSA and cardiovascular diseases is related to neural activation, oxidative stress, systemic inflammation, and physiological hormone disturbance caused by hypoxemia (71–73). Studies have shown that C-reactive protein is an independent risk factor for atrial fibrillation, and OSA can increase C-reactive protein levels (74). Hypoxic reperfusion leads to vascular endothelial dysfunction (71), excessive release of sympathetic vasoconstrictor substances, and decreased nitric oxide bioavailability leading to elevated blood pressure (75). In cardiovascular events, the risk of Aortic dissection, MACEs and essential arterial hypertension increases with the severity of OSA.

Patients with OSA are at risk for serious eye disease, and the meta-analysis we included demonstrated that OSA is strongly associated with multiple eye diseases (16, 17). We included the largest study of all meta-analyses showing a 49% increased risk of developing glaucoma in OSA patients. Among them, hypoxia-induced optic papillary ischemia, hemodynamic changes of retinal blood vessels, oxidative stress inflammation, and mitochondrial dysregulation lead to nerve fiber dysfunction that contributes to the occurrence of glaucoma (76). Relevant studies have shown that the activity of proteolytic enzyme matrix metalloproteinases (MMPs) to degrade extracellular mechanisms is higher during hypoxic stress or injury, and we found an increase of MMP-9 in the tears of patients with keratoconus, which contributes to the thinning of the cornea (77). In the meta-analysis we included, we found that long-term chronic hypoxia in OSA patients leads to nerve activation, oxidative stress, systemic inflammation, and physiological hormone disorders, which lead to the occurrence of disease, which is the mechanism of the association between OSA and most diseases. Nasal continuous positive airway pressure (CPAP) is the standard treatment for patients with moderate to severe obstructive sleep apnea. It can keep the upper respiratory tract open, thus alleviating hypoxemia in the body and reducing the occurrence of various body reactions. At the same time, lifestyle interventions (exercise, weight loss, low-fat diet, smoking and alcohol cessation, et al.) are also necessary (78).

Notably, this is the first comprehensive and systematic review to assess the potential relationship between OSA and multiple adverse clinical outcomes. With the increasing incidence of OSA in the population, this umbrella review has important clinical implications for developing strategies for early detection, diagnosis, and treatment of OSA, and even for identifying the cause of the disease. Umbrella reviews provide a comprehensive summary of published meta-analyses and are an effective tool for studying the relationship between complex adverse clinical outcomes and variables. Depending on the type of meta-analysis included, the effect size and 95% confidence interval were re-evaluated using either a fixed-effects model or a random-effects model, and the heterogeneity and publication bias of each included meta-analysis were assessed. We also used the AMSTAR and GRADE evaluation tools to assess the quality and level of evidence for each of the included adverse clinical outcomes. As a whole, most of the included meta-analyses have high heterogeneity and low-quality evidence, and larger and higher-quality studies are needed to further explain the relationship between them.

Although we have optimized our screening strategy, there are still some limitations. First, due to the particularity of the research, most of the meta-analyses included retrospective studies, which inevitably leads to the low quality evidence of the meta-analysis. In some case–control studies, recall bias is unavoidable. Secondly, most OSA patients are combined with other health problems (obesity, diabetes), and there are many confounding factors affecting adverse clinical outcomes, large heterogeneity is observed, and there are some adverse clinical outcomes with insignificant associations. Obstructive ventilation disorders occur in OSA patients mostly during sleep at night, and most OSA is not detected. Finally, the impact of short-term or long-term, persistent status of OSA on adverse clinical outcomes is also a complex issue, and for some diseases, there is a gap in outcome performance if follow-up time is insufficient. In future large studies, we should use more precise methods to diagnose OSA, and more studies should provide data quantifying exposure levels to enable more accurate dose–response analysis.

## Conclusion

In summary, given the rising prevalence of OSA in recent years, the associated clinical complications have also increased. Therefore, early identification and intervention for OSA are critically important. However, the quality of evidence from multiple studies is low, and larger cohort studies or randomized controlled trials are needed for further proof. To address the high incidence of OSA and its association with adverse outcomes, a combination of extensive health education and policies worldwide is urgently needed.

## Data Availability

The original contributions presented in the study are included in the article/Supplementary material, further inquiries can be directed to the corresponding author.

## Supplementary material

SUPPLEMENTARY TABLE 1 The AMSTAR and GRADE evaluation of included meta-analyses. AMSTAR, a measurement tool to assess systematic reviews; GRADE, Grading of Recommendations Assessment, Development, and Evaluation.

## Glossary

OSA: Obstructive sleep apnea
AHI: Apnea-hypopnea index
RR: Relative risk
OR: Odds ratio
HR: Hazard risk
AMSTAR: A measurement tool to assess systematic reviews
GRADE: Grading of recommendations, assessment, development, and evaluation
95% CI: 95% confidence interval
OSAS: Obstructive sleep apnea syndrome
OSASH: Obstructive sleep apnea-hypopnea Syndrome
GERD: Gastroesophageal reflux disease
NAFLD: Non-alcoholic fatty liver disease
AF: Atrial fibrillation
FES: Floppy eyelid syndrome
LEC: Lax eyelid condition
RVO: Retinal vein occlusion
CSR: Central serous chorioretinopathy
IIH: Idiopathic intracranial hypertension
NICU: Neonatal intensive care unit
MACEs: Major adverse cardiac events
FRC: Functional residual capacity
VEGF: Vascular endothelial growth factor
GLUT-1: Glucose transporter 1
NSCLC: Non-small cell lung cancer
MMPs: Matrix metalloproteinases
CPAP: Continuous positive airway pressure
